# Rapid Determination of Vitamin D_3_ in Aquatic Products by Polypyrrole-Coated Magnetic Nanoparticles Extraction Coupled with High-Performance Liquid Chromatography Detection

**DOI:** 10.3390/nano12071226

**Published:** 2022-04-06

**Authors:** Xinyan Liu, Ru Song, Rongbian Wei

**Affiliations:** 1 Key Laboratory of Health Risk Factors for Seafood of Zhejiang Province, School of Food Science and Pharmacy, Zhejiang Ocean University, Zhoushan 316022, China; 18742055080@163.com; 2 School of Chemistry and Bioengineering, Guangxi Normal University for Nationalities, Chongzuo 532200, China; apwapw@126.com

**Keywords:** Fe_3_O_4_ nanoparticles, pyrrole, characteristics, vitamin D_3_, adsorption, aquatic products

## Abstract

A method using polypyrrole-coated Fe_3_O_4_ (Fe_3_O_4_@PPy composites) based extraction coupled with high performance liquid chromatography was developed for adsorption and detection of trace vitamin D_3_ (VD_3_) in aquatic products. The fabricated Fe_3_O_4_@PPy composites were characterized by scanning electron microscopy, transmission electron microscopy, X-ray diffraction, Fourier transform infrared spectroscopy, and thermogravimetric analysis. Fe_3_O_4_@PPy composites showed efficient adsorption of VD_3_ at pH 9.0 and 25 °C with a dose of 25 mg per 10 mL of sample solution and an adsorption time of 11 min. Methanol was selected as the desorption solvent to recover VD_3_ from Fe_3_O_4_@PPy composites after 3 min of static treatment. Fe_3_O_4_@PPy composites can be used for VD_3_ adsorption at least two times. The developed method showed a good linearity for VD_3_ determination in the range of 0.1–10 μg/mL with a correlation coefficient of 0.9989. The limits of detection and quantification were 10 ng/mL and 33 ng/mL, respectively. The recovery of VD_3_ in a spiking test was 97.72% with a relative standard deviation value of 1.78%. The content of VD_3_ in nine aquatic products was determined with this method. Our results show that Fe_3_O_4_@PPy composites provide a convenient method for the adsorption and determination of VD_3_ from the complex matrix of aquatic products.

## 1. Introduction

Vitamin D (VD), a group of fat-soluble secosteroids, plays important roles in the physiological activity of humans. The traditional role of VD is to maintain calcium and phosphorus homeostasis and normal bone function and structure [1]. Recently, more and more studies have reported that various chronic diseases, including insulin resistance, diabetes, and cardiovascular disease are linked to a VD deficiency [2,3]. In nature, VD mainly exists in two physiological forms based on different side-chains. Ergocalciferol (VD_2_) is mainly found in plants and cholecalciferol (VD_3_) comes from animals [4]. It is considered that fatty fish, fish liver, and fish oil are excellent supplementation sources for natural VD_3_ [5,6]. Other foods, such as meat and egg yolk, also contain high amounts of VD_3_ [5].

The complexity of food matrices means that measuring the content of VD_3_ needs appropriate pretreatment before instrumental analysis. Appropriate extraction techniques can remove substances, including proteins, polysaccharides, and lipids that can interfere with VD_3_ detection, and therefore, enhance the accuracy and detection limit of the method [7]. Popular extraction techniques are liquid-liquid extraction, solid phase extraction (SPE), dispersive liquid-liquid microextraction, magnetic solid phase extraction (MSPE), and enzyme linked immunosorbent assay [8,9,10]. Considering the amount of organic solvent consumption, environmental friendliness, adsorbent usage, and time or cost [3,11], the MSPE technique has attracted great attention due to the rapid separation of the adsorbent from the sample matrix with a magnetic field [12]. Among the various magnetic nanoparticles, Fe_3_O_4_ nanoparticles have many unique properties (i.e., low toxicity, biocompatibility, and biodegradability) [13] and have been applied in various fields, such as heavy metal removal from industrial wastewater [14], trace component enrichment before detection [15,16], adsorption of target biomedical compounds [17], etc. However, Fe_3_O_4_ nanoparticles can be easily oxidized and can form aggregates [18]. Therefore, it is necessary to modify the surface of Fe_3_O_4_ nanoparticles to improve their stability.

Polypyrrole (PPy), the polymer of monomeric pyrrole, offers the advantages of low toxicity, low cost, and easy preparation [19]. PPy has been used on Fe_3_O_4_ nanoparticles to form an adhesive coat with more binding sites for target molecules by means of π-π stacking, hydrogen bonding, van der Waals forces, and charge interactions [20]. Fabricated Fe_3_O_4_@PPy composites have been successfully utilized for the removal of Ni (II) and Cr (VI) from industrial effluent [21] and rapid extraction of antiseptic ingredients or estrogens before HPLC-MS/MS determination [22,23]. In a recent study, Jiao, Zhang and Fan (2016) [10] described the effective extraction capacity of Fe_3_O_4_@PPy for vitamin D_2_ and vitamin D_3_ in the milk matrix. Although PPy coated Fe_3_O_4_ nanoparticles have been successfully prepared in the above literature, their structures and properties may be varied due to different synthesis methods. Moreover, the adsorption capacity of Fe_3_O_4_@PPy for different molecules, or even the same molecule, may vary in different food matrices. As far as we know, there are few reports on the application of Fe_3_O_4_@PPy for the extraction of VD_3_ from aquatic products before detection.

The aim of this work was to prepare a suitable Fe_3_O_4_@PPy composite with a high adsorption efficiency for trace VD_3_ in aquatic products before detection by high performance liquid chromatography (HPLC). The fabricated Fe_3_O_4_@PPy composites were characterized by scanning electron microscopy (SEM), transmission electron microscopy (TEM), X-ray diffraction (XRD), Fourier transform infrared spectroscopy (FTIR), and thermogravimetric analysis (TGA) measurements. Furthermore, the main parameters affecting the efficiency of extraction, including sample pH, adsorbent dose, adsorption temperature, adsorption time, desorption solvent, and desorption time were optimized or determined. Finally, the method of Fe_3_O_4_@PPy extraction coupled with HPLC for detection of VD_3_ was evaluated and employed for nine aquatic products. Our results provide an efficient, rapid, and environmentally friendly method for the detection of trace VD_3_ in aquatic product matrices.

## 2. Materials and Methods

### 2.1. Materials

The aquatic products used in this study, including Penaeus sinensis (*Solenocera crassicornis*), Pacific white shrimp (*Litopenaeus vannamei*), cuttlefish (*Sepia esculenta*), squid (*Loliolus japonica*), clams (*Cyclina sinensis* and *Paphia undulata*), razor clam (*Sinonovacula constricta*), Chlamys farreri (*Azumapecten farreri*), and silver pomfret (*Pampus argenteus*) were purchased from a local market in Zhoushan City, transported to the laboratory with ice bags within 1 h, and stored at −20 °C for no more than one month. Vitamin D_3_ standard (VD_3_) (purity > 98%) and pyrrole were purchased from Aladdin Industrial Corporation (Shanghai, China). Chemicals used for HPLC analysis were HPLC grade and purchased from Sinopharm Chemical Reagent Co., Ltd. (Shanghai, China). All other reagents were of analytical grade and obtained from commercial products.

### 2.2. Sample Saponification

All of the aquatic products used in this study were saponified to release VD_3_ from bound protein using the method of Strobel, Buddhadasa, Adorno, Stockham and Greenfield (2013) [24] with further modifications. In brief, 4.0 g of minced sample was added to 10 mL of an 80% ethanol solution (containing 20% KOH), followed by the addition of 20.0 mg ascorbic acid. After 2 h of oscillation at room temperature, the mixture was centrifuged for 10 min at 6000 rpm. Finally, the supernatant was collected and used for the VD_3_ adsorption experiments.

### 2.3. VD_3_ Concentration Measurement

VD_3_ concentration was determined by de Azevedo’s method [25] with slight modifications. Briefly, 10 μL of sample filtrate was loaded onto a 1260 Agilent HPLC system (Waldbronn, Germany) with a C_18_ column (4.6 × 250 mm, 5 μm) (Elite, Dalian, China) at 40 °C. The mobile phase was 100% methanol with a flow rate of 0.6 mL/min and detection at 264 nm. To calculate sample VD_3_ concentration, VD_3_ standards were prepared in methanol with concentrations ranging from 0 to 10.0 μg/mL and assayed under the same conditions. VD_3_ concentration was determined from the calibration curve for the VD_3_ standards (y = 15.38 × −0.0583, R^2^ = 0.9975).

### 2.4. Preparation of Fe_3_O_4_@Polymerization of Pyrrole (Fe_3_O_4_@PPy)

#### 2.4.1. Fe_3_O_4_ Nanoparticles (Fe_3_O_4_ NPs) Preparation

Fe_3_O_4_ NPs were prepared according to the method of Nalle, Wahid, Wulandari and Sabarudin (2019) [26] with slight modifications. In brief, 0.18 g of FeCl_2_·4H_2_O and 0.3 g of FeCl_3_·6H_2_O were dissolved in 15 mL of deionized water degassed by ultrasonic treatment, and stirred for 15 min at 55 °C. Then, 7.2 mL of 3 mol/L NaOH was rapidly added to the mixture and continuously stirred for 40 min. After 30 min of incubation at 90 °C in a water bath, the reaction solution was cooled to room temperature. The resulting black sediment was washed repeatedly with deionized water until a pH of 7.0 was achieved, then dried in a vacuum oven at 60 °C overnight. The generated Fe_3_O_4_ NPs were stored for further experiments.

#### 2.4.2. Synthesis of Fe_3_O_4_@PPy Composites

Fe_3_O_4_ NPs were coated by polymerization of pyrrole (PPy) using the method of Zhang et al. (2020) [23] with few modifications. In brief, 0.028 g of sodium dodecyl sulfate and 0.2 g of Fe_3_O_4_ were added to 80 mL of deionized water and sonicated for 20 min to obtain a homogeneous dispersion. Subsequently, pyrrole monomer at a ratio of 1:1, 3:1, 5:1 (*v*/*w*) with respect to Fe_3_O_4_ NPs content was added and stirred for 10 min. Then, 10 mL of 1 mol/L FeCl_3_·6H_2_O was added slowly to the reaction and stirred for 12 h at room temperature. The generated Fe_3_O_4_@PPy particles coated with different PPy ratios were recovered using an external magnetic field, washed with deionized water three times, and finally dried in a vacuum oven at 60 °C overnight. The VD_3_ adsorption rate and particle size of these freeze dried Fe_3_O_4_@PPy were then determined.

### 2.5. Adsorption Rate for VD_3_

Fe_3_O_4_@PPy powder (50 mg) was added to a saponified solution (10 mL resulting from 4 g of Penaeus sinensis by-products). After 20 min of static adsorption at room temperature, the Fe_3_O_4_@PPy composites were separated from the mixture using an external magnet, washed with 2 mL of deionized water and 2 mL of ethanol. Then, the collected Fe_3_O_4_@PPy particles were added to 2 mL of methanol and desorbed for 10 min in a standing state, followed by separation of the Fe_3_O_4_@PPy particles using the action of a magnet. The remaining solution was evaporated at 45 °C to remove the methanol, and then redissolved in 500 μL of methanol. After filtration through a 0.22 μm filter, the concentration of VD_3_ was determined as described in Section 2.3. The adsorption rate of VD_3_ was calculated according to Equation (1) as follows:

Adsorption rate/% = (c × v)/m_0_ (1)

where c represents the concentration of VD_3_ (μg/mL), v represents the total volume of the filtrate (mL), and m_0_ (μg) represents the theoretical amount of VD_3_ extracted with liquid-liquid extraction (LLE) using hexane as the solvent, calculated by multiplying the concentration of VD_3_ in the LLE by the volume. The Fe_3_O_4_@PPy composites fabricated with the ratio of PPy that showed the highest adsorption rate of VD_3_ were used for further experiments.

### 2.6. Particle Size Measurement

The Fe_3_O_4_@PPy composites prepared with different ratios of PPy (1:1, 3:1, 5:1, *v*/*w*) were dispersed by sonication in pure water for 10 min. Then, 1.0–1.5 mL of the dispersion was dropped into the sample pool of a Zeta-sizer Nano-ZS90 (Malvern Instruments, Worcestershire, UK). The mean particle diameter (z-average) was determined in triplicate at 25 °C.

### 2.7. Characterization of Fe_3_O_4_@PPy Composites

#### 2.7.1. Scanning Electron Microscopy (SEM) and Transmission Electron Microscope (TEM)

Dried Fe_3_O_4_@PPy particles were characterized by SEM (JSM-7800F, JEOL, Japan) with an acceleration voltage of 10.00 kV, working distance of 6.1 mm, magnification at 50.00 KX, and signal A from the in-lens detector. A droplet of the Fe_3_O_4_@PPy composites suspension in distilled water was placed on a carbon coated copper mesh, and the excess liquid was removed with filter paper. Then, the Fe_3_O_4_@PPy particles were observed using TEM (Tecnai G2 F30, FEI, HILLSBORO, OR, USA) under an appropriate magnification. Corresponding SEM and TEM images of Fe_3_O_4_ NPs were used for comparison.

#### 2.7.2. Fourier Transform Infrared (FTIR) Analysis

Dried Fe_3_O_4_@PPy composites or Fe_3_O_4_ NPs were ground with dried KBr at 1:100 (m/m), pressed into a thin slice, and then recorded with an FTIR spectrometer (IRAffinity, Shimadzu, Japan) from 400–4000 cm^−1^.

#### 2.7.3. X-ray Diffraction (XRD) Analysis

XRD patterns for the Fe_3_O_4_@PPy composites or Fe_3_O_4_ NPs were determined by an X-ray diffractometer (MiniFlex600, Rigaku, Japan) using Cu-Kα radiation in the region of 2θ from 10° to 70° at a scanning rate of 0.02°/s.

#### 2.7.4. Thermogravimetric Analysis (TGA)

The thermogravimetric property of the Fe_3_O_4_@PPy composites was measured using a thermogravimetric analyzer (DTG-60, Shimadzu, Japan) under a N_2_ atmosphere at temperatures ranging from room temperature to 600 °C. Fe_3_O_4_ NPs were assayed in parallel for comparison.

### 2.8. Adsorbent Experiment

#### 2.8.1. Adsorption Conditions

The adsorption experiments were carried out in a 100 mL conical flask containing 10 mL of the VD_3_ saponification solution at various pHs (6.0–14.0) with a range of adsorbent doses (0.005–0.05 g Fe_3_O_4_@PPy composites), adsorption temperatures (25–55 °C), and adsorption times (3–30 min). After adsorption, the Fe_3_O_4_@PPy composites were collected from the mixture with an external magnet. Subsequently, VD_3_ desorption from Fe_3_O_4_@PPy composites was carried out using methanol as the desorption solvent according to the conditions described in Section 2.5. The concentration of VD_3_ was determined by HPLC as described in Section 2.3. The amount of VD_3_ adsorbed to the Fe_3_O_4_@PPy composites was calculated as q_e_ (μg/g) using Equation (2) as follows:

q_e_ = (c × v)/m_p_ (2)

where c represents the concentration of VD_3_ (μg/mL), v represents the total volume of the filtrate (mL), and m_p_ is the mass of the Fe_3_O_4_@PPy composites used in the experiment (g).

#### 2.8.2. Desorption Conditions

To develop a satisfactory desorption method, the desorption solvent and desorption time were investigated further. After VD_3_ adsorption, the collected Fe_3_O_4_@PPy composites were added to 2.0 mL of desorption solvent (ethanol, acetonitrile, and methanol) and sonicated or allowed to stand for 1 to 15 min at room temperature. After magnetic separation of the Fe_3_O_4_@PPy composites, the resulting solution was evaporated and then redissolved in methanol as described in Section 2.4.2. Finally, 10 μL of filtrate was analyzed for VD_3_ content using HPLC as described in Section 2.3. The desorption rate of VD_3_ from Fe_3_O_4_@PPy composites was calculated according to Equation (3) as follows:

Desorption rate/% = (c × v)/m_a_ (3)

where c represents the VD_3_ concentration (μg/mL), v represents the total volume of the filtrate (mL), and m_a_ is the total amount of VD_3_ adsorbed by the Fe_3_O_4_@PPy composites (μg).

### 2.9. Reusablility of Fe_3_O_4_@PPy Composites

To investigate the recyclability of the Fe_3_O_4_@PPy composites as adsorbents for VD_3_ in saponified aquatic products, one batch of Fe_3_O_4_@PPy composites was used as the adsorbent to conduct the adsorption and desorption experiments. The recovery of VD_3_ from recycled Fe_3_O_4_@PPy composites was compared after repeated use. In addition, the characteristics of recycled Fe_3_O_4_@PPy composites were determined using SEM, TEM, XRD and FTIR measurements.

### 2.10. Method Evaluation

Quantitative parameters for the HPLC determination of VD_3_ after Fe_3_O_4_@PPy composites extraction, including linearity, coefficient of determination (r^2^), limits of detection (LOD), limits of quantification (LOQ), accuracy and precision, were evaluated under optimal adsorption and desorption conditions. The sensitivity of the method was evaluated by the LOD and LOQ at a signal-to-noise ratio of 3 (S/N = 3) and 10 (S/N = 10), respectively. The accuracy of recovery was assessed by spiking saponified samples with a VD_3_ standard (2 μg) and calculating recovery according to Equation (4). These samples were analyzed six times per day and the precision of the method was evaluated by intra-day relative standard deviation (RSD).

Recovery/% = (Detected amount − sample amount)/Standard added amount × 100(4)

### 2.11. Application of Fe_3_O_4_@PPy Composites for VD_3_ Detection in Aquatic Products

Aquatic products, mentioned in 2.1., were homogenized and saponified as described in Section 2.2. After the pH of the saponified sample solution was adjusted to 9.0, 25 mg of the Fe_3_O_4_@PPy composites were added for VD_3_ extraction. After standing at room temperature (25 °C) for 11 min, the adsorbent was separated from the mixture using magnets. The adsorbent was rinsed with 2.0 mL of deionized water and then desorbed statically with 2.0 mL of methanol for 3 min. This methanol solution was magnetically separated from the Fe_3_O_4_@PPy particles and evaporated to dryness using a vacuum rotary evaporator, then redissolved in 500 μL of methanol. Finally, the methanol solution was filtered through a 0.22 μm organic filter, and 10 μL of the filtrate was analyzed by HPLC for VD_3_ determination. The amount of VD_3_ in aquatic products was expressed as μg per 100 g.

### 2.12. Statistic Analysis

All experimental results were expressed as the mean ± standard deviation. Analyses were performed with a one-way analysis of variance (ANOVA) and Tukey’s test using SPSS^®^ software 19.0 (Chicago, IL, USA) to determine significant differences at *p* ≤ 0.05.

## 3. Results and Discussion

### 3.1. Effect of PPy to Fe_3_O_4_ NPs Ratio on the Adsorption Rate of VD_3_

**Figure 1:**
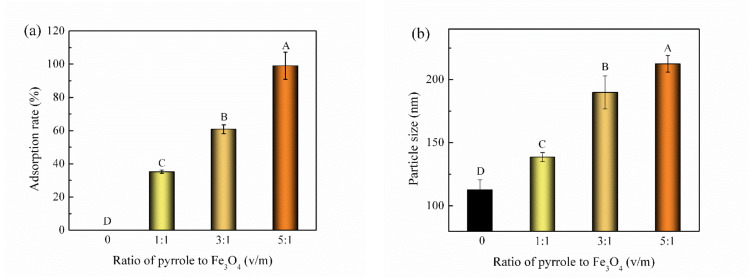
Effect of the ratio of pyrrole to Fe_3_O_4_ on the adsorption rate for VD_3_. (**a**) Adsorption rate and (**b**) particle size of Fe_3_O_4_@PPy composites. Fe_3_O_4_ NPs were used for comparison (ratio of 0). Data is expressed as the mean ± standard deviation (*n* = 3). The different capital letters (A–D) indicate a significant difference (*p* < 0.05).

As is shown in Figure 1a, the VD_3_ adsorption rate for the Fe_3_O_4_@PPy composites increased with the dose of PPy. At ratio of 5:1 for PPy and Fe_3_O_4_ (*v*/*m*), the adsorption rate for VD_3_ reached 100% (*p* < 0.05). The particle size of Fe_3_O_4_@PPy composites also increased with the amount of PPy added (Figure 1b). This indicated that an increase in pyrrole monomer led to a thickening of the coating on the surface of Fe_3_O_4_ NPs and provided more binding sites, thus improving the adsorption of VD_3_. In this study, Fe_3_O_4_@PPy composites prepared at ratio of 5:1 (pyrrole/Fe_3_O_4_, *v*/*w*) had the highest adsorption rate for VD_3_, and therefore, were selected for subsequent experiments.

### 3.2. Characterization of Fe_3_O_4_@PPy Composites

#### 3.2.1. SEM and TEM Observation

**Figure 2:**
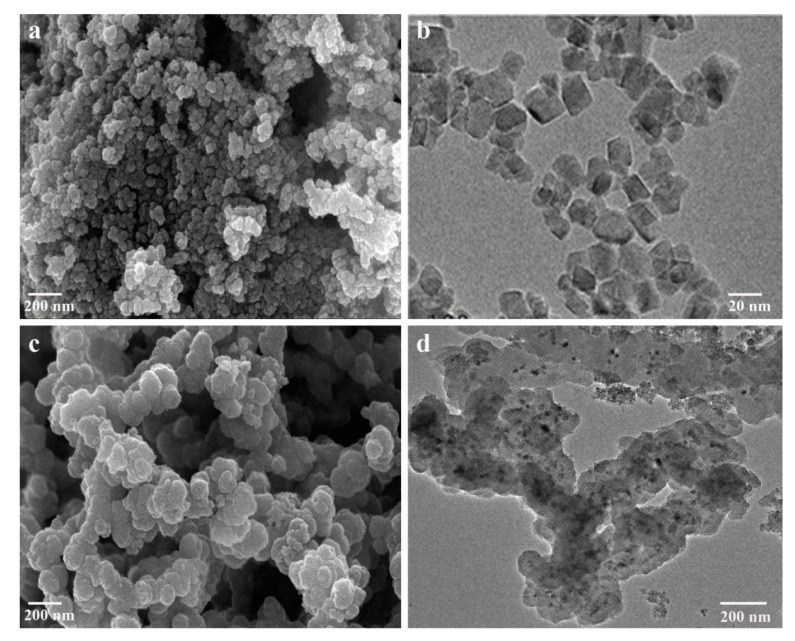
Microstructure of the Fe_3_O_4_@PPy composites under SEM and TEM. (**a**) SEM image of Fe_3_O_4_ NPs. (**b**) TEM image of Fe_3_O_4_ NPs. (**c**) SEM image of Fe_3_O_4_@PPy composites. (**d**) TEM image of Fe_3_O_4_@PPy composites.

Both Fe_3_O_4_ NPs and Fe_3_O_4_@PPy composites comprised clusters of spherical particles when observed with SEM (Figure 2a,c). The co-ion effect of the Fe^3+^ oxidant is considered the main reason for the aggregation of Fe_3_O_4_ NPs [18] and a similar phenomenon has also been reported for the complexation of Fe^3+^ with PPy [23]. When viewed with TEM, the Fe_3_O_4_ NPs and Fe_3_O_4_@PPy composites had a different appearance. As is shown in Figure 2b, Fe_3_O_4_ NPs had a smooth and uniform surface morphology with diameters from 10 to 20 nm. By comparison, the image in Figure 2d shows that a PPy shell had been successfully coated onto the surface of the Fe_3_O_4_ NPs, where the clearly black regions are related to the Fe_3_O_4_ NPs at the core surrounded by light regions that have been formed by the polymerization of pyrrole monomers in the outer layer [27].

#### 3.2.2. XRD Analysis

**Figure 3:**
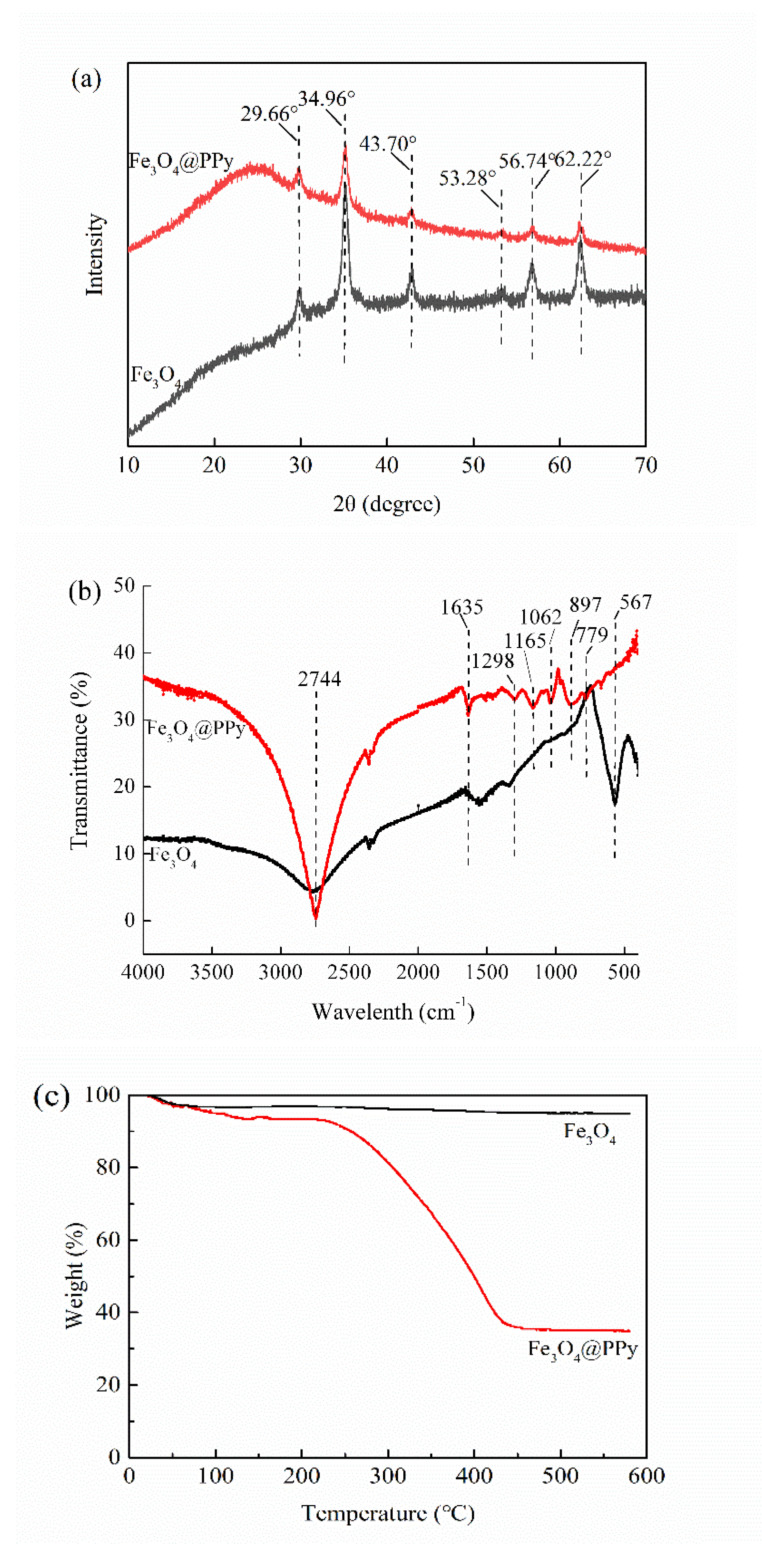
Characteristics of the Fe_3_O_4_@PPy composites compared with Fe_3_O_4_ NPs. (**a**) XRD pattern, (**b**) FTIR spectra, and (**c**) TGA curves.

The Fe_3_O_4_ NPs prepared in this study showed typical peaks of XRD at 2θ of 29.66°, 34.96°, 43.70°, 53.28°, 56.74°, and 62.22° (Figure 3a), which were accordance with previous reports on Fe_3_O_4_ NPs [28]. After coating with PPy, the XRD pattern of the Fe_3_O_4_@PPy composites had similar peaks to those detected in Fe_3_O_4_ NPs, indicating the presence of Fe_3_O_4_ NPs. Similar results were reported for the characteristics of iron oxides in Fe_3_O_4_@PPy [10]. However, the intensities of these peaks were all decreased to some extent. Furthermore, a broad peak was observed in the low range of 20–30°, which was ascribed to the typical amorphous structure of polypyrrole [21]. The findings in Figure 3a further provide further evidence of the existence of a PPy coating on the surface of the Fe_3_O_4_ NPs, consistent with TEM images (Figure 2d).

#### 3.2.3. FTIR Analysis

The FTIR spectra of the Fe_3_O_4_ NPs and Fe_3_O_4_@PPy composites are compared in Figure 3b. The typical band at 567 cm^−1^ resulted from the stretching vibration of the Fe–O bond in Fe_3_O_4_ [29]. After interaction with PPy, the bands at 779 cm^−1^ and 897 cm^−1^ related to =C–H out-of-plane vibration of pyrrole rings [18] were observed in the Fe_3_O_4_@PPy composites. Furthermore, some of the typical bands associated with PPy, such as 1298 cm^−1^ (C–H in-plane vibration), 1165 cm^−1^ (=C–H in plane vibration), and 1062 cm^−1^ (C-N stretching vibration) [30,31] were detected in the Fe_3_O_4_@PPy composites. The appearance of a band at 1635 cm^−1^ might be ascribed to the red shift of basic C=C stretching of the Py ring due to slight over-oxidation [27,31]. Additionally, the band at 2744 cm^−1^ related to C–H stretching vibration was dramatically increased in the Fe_3_O_4_@PPy composites. All the typical bands corresponding to PPy, as well as the missing band at 567 cm^−1^, suggest that the Fe_3_O_4_ NPs were enveloped by a PPy coating.

#### 3.2.4. TGA

Figure 3c shows the TGA curves for Fe_3_O_4_ NPs and Fe_3_O_4_@PPy composites. The weight loss from Fe_3_O_4_ NPs was 3.02% after heating from 25 °C to 100 °C, which was related to the evaporation of a small amount of water. The weight of Fe_3_O_4_ NPs (94.96%) was stable at temperatures above 100 °C. By comparison, three stages were detected in the TGA pattern for the Fe_3_O_4_@PPy composites. In the first stage, the evaporation of water and a slight degradation of PPy could be responsible for the observed weight loss of 6.58% when Fe_3_O_4_@PPy was heated from 100 °C to 200 °C. In the second stage, the Fe_3_O_4_@PPy composites decomposed dramatically at about 250 °C, which is consistent with the TGA results for a Fe_3_O_4_-PPy composite with a Fe_3_O_4_ content of 34% described by Chen et al. (2003) [30]. In the third stage, the TGA pattern for Fe_3_O_4_@PPy showed that 34.87% of the core content (Fe_3_O_4_) was left behind at 450 °C. The results in Figure 3c should be sufficient to prove that the fabricated Fe_3_O_4_@PPy composites have a core-shell structure.

### 3.3. Conditions for VD_3_ Adsorption to Fe_3_O_4_@PPy Composites

#### 3.3.1. Effect of pH

**Figure 4:**
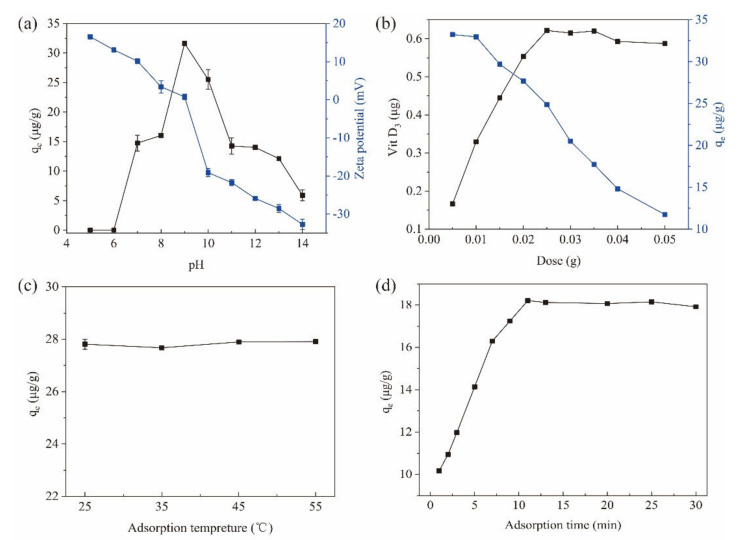
Effects of factors on the adsorption of VD_3_ by Fe_3_O_4_@PPy. (**a**) pH value of sample, (**b**) adsorbent dose, (**c**) adsorption temperature, and (**d**) adsorption time. Data are expressed as the mean ± standard deviation (*n* = 3).

The pH value of the adsorption environment is a critical factor in MSPE methods, since it can change the surface net charges for both the magnetic materials and the target compounds [23]. In this study, the initial pH of the sample solution (saponified Penaeus sinensis by-products) was 14.0. By decreasing pH, the adsorption (q_e_) of VD_3_ increased from 5.91 μg/g at pH 14.0 to 31.64 μg/g at pH 9.0 for the Fe_3_O_4_@PPy composites (Figure 4a). However, when the pH was lowered further, the adsorption capacity decreased sharply and was reduced to zero at pH 6 and pH 5. These findings indicate that an acidic environment is not suitable for the adsorption of VD_3_ to the Fe_3_O_4_@PPy composites. Under acidic conditions, some proteins with an isoelectric point of about 5.0 present in saponified aquatic products could precipitate, which might trap the originally released VD_3_, thus resulting in poor adsorption of VD_3_ to Fe_3_O_4_@PPy.

To reveal the role of pH on the adsorption capacity of the Fe_3_O_4_@PPy composites, the zeta potential was measured at different pH values. As shown in Figure 4a, the Fe_3_O_4_@PPy composites were negatively charged when pH conditions were greater than 9.0 (zeta potential < 0). In contrast, the Fe_3_O_4_@PPy composites were positively charged when the pH was below 9.0 (zeta potential > 0). It should be noted that the Fe_3_O_4_@PPy composites had a net charge close to neutral at pH 9.0 and demonstrated the highest adsorption capacity (q_e_) for VD_3_. The results in Figure 4a indicate that the driving force for adsorption of VD_3_ onto the Fe_3_O_4_@PPy composites does not rely on charge interactions, but rather, hydrophobic interactions and/or π-π stacking.

#### 3.3.2. Effect of Adsorbent Dose

To achieve a good adsorption efficacy with a minimal dose of the Fe_3_O_4_@PPy composites, different amounts were applied to extract VD_3_ from saponified Penaeus sinensis by-products. As the adsorbent dose increased from 5 mg to 50 mg, the amount of VD_3_ adsorbed by the Fe_3_O_4_@PPy composites increased with each increase in dosage up to 25 mg, when the maximum adsorption was achieved, and remained stable at higher doses (Figure 4b). Increasing the amount of adsorbent can provide more adsorption sites for the target ingredient, which helps improve the adsorption speed [23]. However, the efficiency of the adsorbent should also be considered because the amount of adsorbent comes at a cost. In this study, the adsorption capacity of the Fe_3_O_4_@PPy composites for VD_3_ decreased gradually with each increase in dose, which could be ascribed to the existence of excess amounts of adsorbent. This could explain why the adsorption dose increased gradually, however the adsorption efficiency did not increase accordingly. Considering the high content of VD_3_ adsorbed and a relatively higher q_e_ compared to other doses, 25 mg was chosen as the optimum dose for the subsequent study.

#### 3.3.3. Effect of Adsorption Temperature

VD is sensitive to heat and degrades easily under high temperatures [32]. The extraction efficiency of VD_3_ from milk by Fe_3_O_4_ @PPy decreased as the extraction temperature increased [10]. However, in this study, when the adsorption temperature ranged from 25 °C to 55 °C, the adsorption capacity of the Fe_3_O_4_@PPy composites for VD_3_ remained stable (Figure 4c). A difference in the sample matrix between the saponified aquatic products used in this study and the unsaponifiable milk samples in the literature might explain the differential effect of temperature on VD_3_ adsorption. Similar to our results, Zhang et al. (2020) [23] described that temperature had little effect on the extraction of 11 antiseptic ingredients with Fe_3_O_4_@PPy composites. Considering the energy saving and a convenient operation, 25 °C was selected as the adsorption temperature for subsequent experiments.

#### 3.3.4. Effect of Adsorption Time

According to previous studies, compared to a conventional SPE method, a quicker equilibrium between the target component and Fe_3_O_4_@PPy nanoparticles can be reached as because of their high surface area and short diffusion route [33]. Figure 4d shows that the adsorption capacity of the Fe_3_O_4_@PPy composites for VD_3_ increased rapidly from 0 to 11 min, then remained stable thereafter. This meant that 11 min was sufficient time to adsorbVD_3_ molecules from the sample matrix. A similar adsorption time of 10 min was reported for VD extraction from milk with Fe_3_O_4_@PPy [10]. Therefore, we selected 11 min as the appropriate time for VD_3_ adsorption to Fe_3_O_4_@PPy composites.

### 3.4. Desorption Conditions

**Figure 5:**
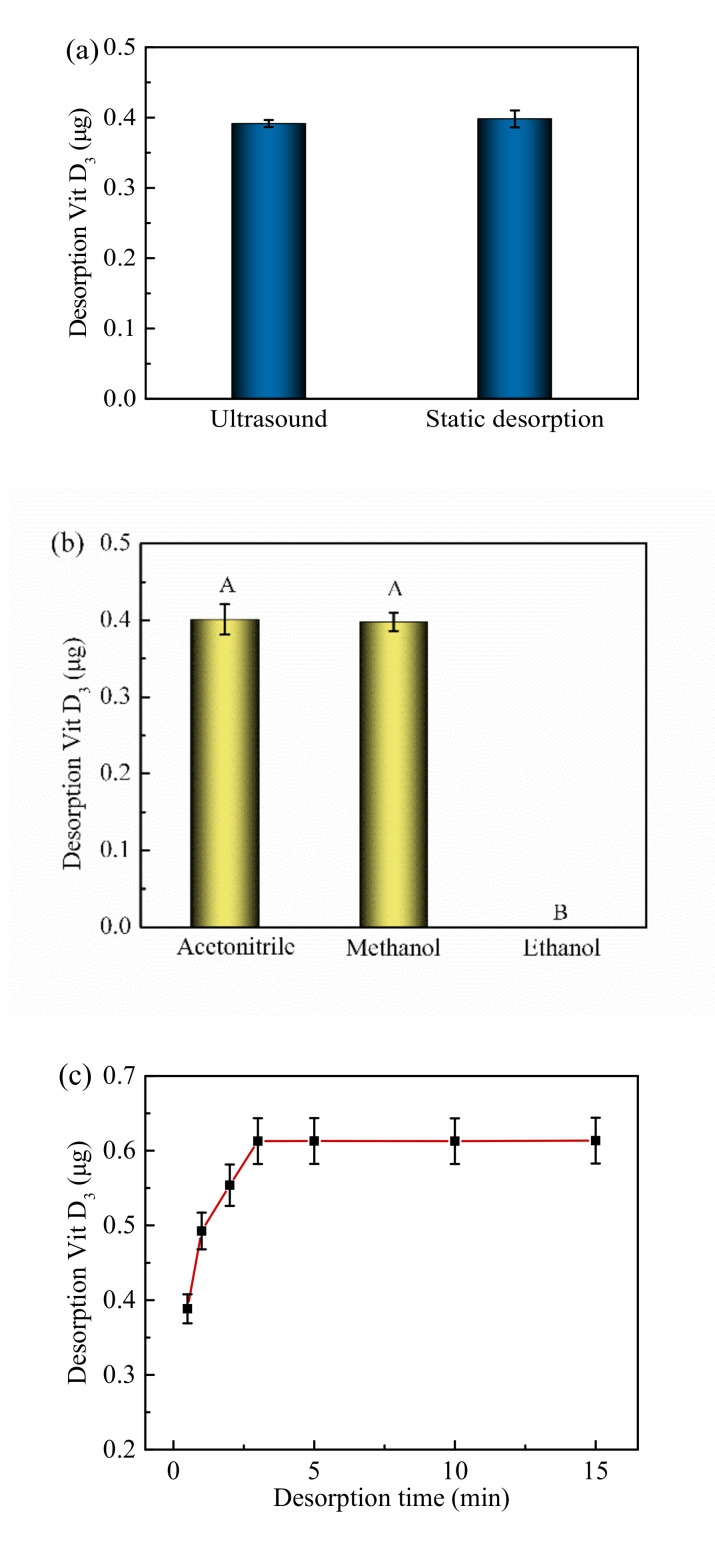
Effects of factors on VD_3_ desorption from Fe_3_O_4_@PPy composites. (**a**) Desorption method, (**b**) desorption solvent, and (**c**) desorption time. Data are expressed as the mean ± standard deviation (*n* = 3). The different capital letters (A,B) indicate a significant difference (*p* < 0.05).

Two desorption methods, namely ultrasonic and static treatment, were used to recover VD_3_ from the Fe_3_O_4_@PPy composites (Figure 5a). No significant difference was found for the two desorption methods (measured as the VD_3_ content in the desorbed solution) (*p* > 0.05). Acetonitrile, methanol, and ethanol were used as desorption solvents under static conditions to recover VD_3_ from the Fe_3_O_4_@PPy composites (Figure 5b). Clearly, acetonitrile and methanol were more efficient than ethanol for desorbing VD_3_ (*p* < 0.05). After a consideration of cost, applicability, and safety, we selected methanol as the desorption solvent. Subsequently, the desorption time with methanol was tested for the Fe_3_O_4_@PPy composites as shown in Figure 5c. The amount of VD_3_ released was found to increase over time from 0.5 min to 3 min, and then remained stable after 3 min. Therefore, we chose 3 min as the desorption time for the follow-up study.

### 3.5. Regeneration of Fe_3_O_4_@PPy Composites

**Figure 6:**
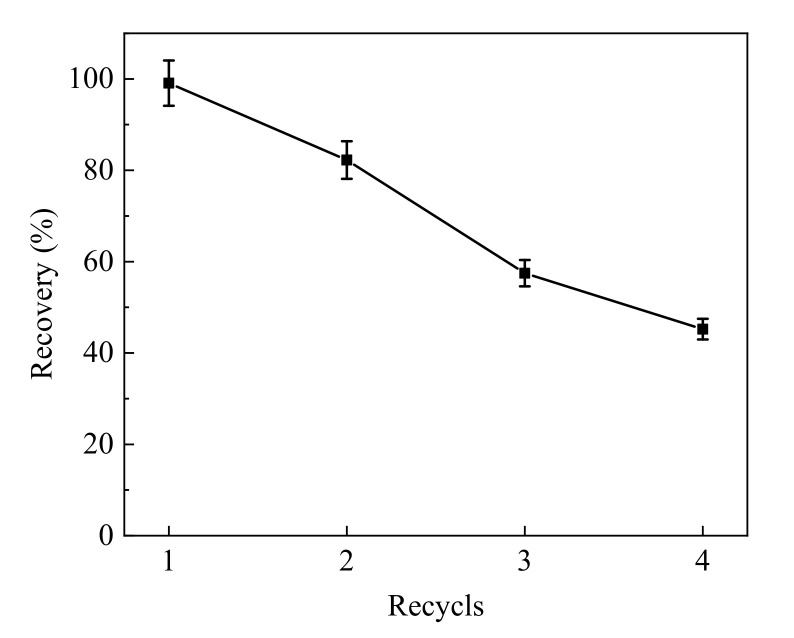
Regeneration experiment using Fe_3_O_4_@PPy composites for VD_3_ adsorption.

To determine if the Fe_3_O_4_@PPy composites prepared in this study could be reused after adsorption of VD_3_ in aquatic products, the performance of the particles was evaluated after repeated adsorption and recovery of VD_3_. After one cycle of adsorption and desorption of VD_3_, the Fe_3_O_4_@PPy composites were dried in a vacuum oven and used for another round of adsorption and desorption. As is shown in Figure 6, the recovery of VD_3_ decreased rapidly each time the Fe_3_O_4_@PPy composites were recycled. At the end of two cycles, the recovery of VD_3_ was 82.25%, but this dropped to 57.48% after three cycles. Our results suggested that the Fe_3_O_4_@PPy composites could be reused at least twice for a VD_3_ recovery over 80%. The decrease in VD_3_ recovery might be due to damage of the PPy shell with repeated use, thus leading to incomplete adsorption of VD_3_.

**Figure 7:**
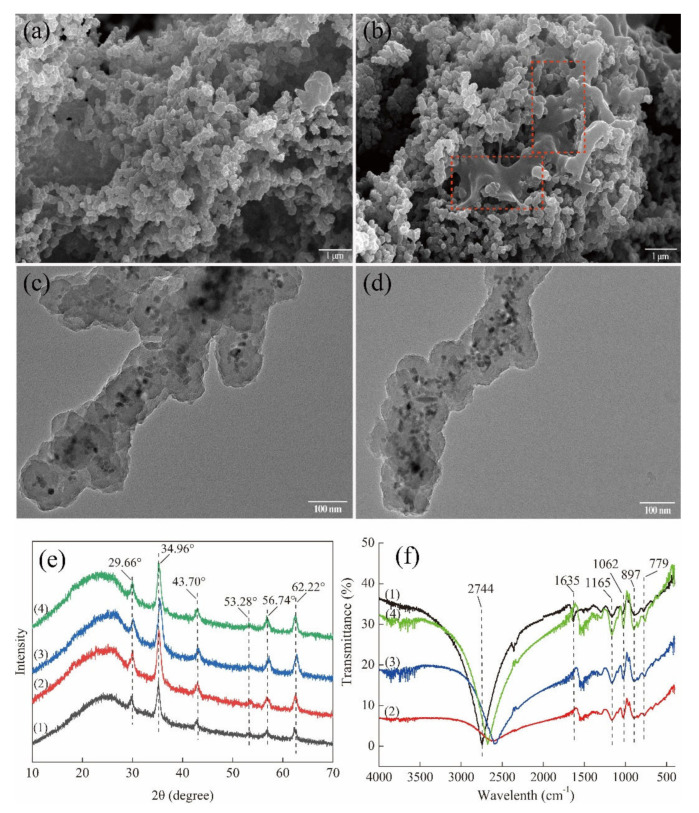
Characteristics of the Fe_3_O_4_@PPy composites after recycling once and twice. SEM images of Fe_3_O_4_@PPy composites reused (**a**) once and (**b**) twice. TEM images of Fe_3_O_4_@PPy composites reused (**c**) once and (**d**) twice. (**e**) XRD and (**f**) FTIR spectra of Fe_3_O_4_@PPy composites. The numbered spectra represent (1) Fe_3_O_4_@PPy, (2) Fe_3_O_4_@PPy after adsorption of VD_3_, (3) Fe_3_O_4_@PPy after the first desorption, and (4) Fe_3_O_4_@PPy after the second desorption.

To investigate possible reasons for the decreased VD_3_ recovery, the characteristics of recycled Fe_3_O_4_@PPy composites were compared using SEM and TEM, as well as XRD and FTIR analysis. No obvious microstructural changes were observed for the recycled Fe_3_O_4_@PPy composites with TEM (Figure 7c,d) when compared to its original condition (as shown in Figure 2d). In contrast, SEM identified some irregular areas wrapped in spherical particles after the composites that had been reused twice (see dotted outlines in Figure 7b), which were not present in unused composites (as shown in Figure 2c) or those that had been recycled once (Figure 7a). These changes might reduce the contact area for VD_3_ adsorption, which might be responsible for the reduction in VD_3_ adsorption.

The specific peaks in the XRD spectrum of the Fe_3_O_4_@PPy composites related to the Fe_3_O_4_ NPs were not affected by adsorption of VD_3_ (Figure 7e). A similar phenomenon was found for the reused magnetic composites. However, the broad peak in the range of 20–30° that was associated with the typically amorphous structure of PPy shifted obviously to smaller angles, suggesting structural changes in the PPy shell after the Fe_3_O_4_@PPy composites had been reused twice. Regarding the FTIR spectra, the band at 1635 cm^−1^ associated with C=C stretching vibration of PPy disappeared from the Fe_3_O_4_@PPy composites after adsorption of VD_3_ (Figure 7f), which implied that the functional C=C group is involved in a hydrophobic interaction between Fe_3_O_4_@PPy and VD_3_. However, the band representing C=C stretching vibration in reused Fe_3_O_4_@PPy composites was not restored after rinsing with methanol (the desorption solvent). This means this functional group might be irreversibly damaged by the desorption solvent. Furthermore, the reused Fe_3_O_4_@PPy composites showed increased intensities for the bands at 1062 cm^−1^, related to C-N stretching vibration, and 1165 cm^−1^, assigned to =C-H in plane vibration [30]. Similarly, the intensities of the bands at 897 cm^−1^ and 779 cm^−1^, attributed to the out-of-plane vibration of the =C-H in pyrrole rings [18], increased each time Fe_3_O_4_@PPy was recycled. In addition, the functional group at 2744 cm^−1^ shifted towards blue after adsorption of VD_3_ compared to a red shift that increased each time the Fe_3_O_4_@PPy composites were reused. These changes suggest that the band related to the C-H stretching vibration in PPy might be crucial for VD_3_ adsorption and desorption. The increases in the intensities of the typical peaks related to the PPy ring indicate that the PPy coating might partially fall off the surface of the Fe_3_O_4_@PPy composites with VD_3_ loading, which cannot be recovered by external magnetic adsorption, thus reducing VD_3_ recovery.

### 3.6. Method Validation and Application

Good linearity for the VD_3_ assay was achieved in the range of 0.1–10 μg/mL in saponified solutions of shrimp by-products with correlation coefficients (r^2^) reaching 0.9989. The limit of detection (LOD) (S/N = 3) and the limit of quantification (LOQ) (S/N = 10) were 10 ng/mL and 33 ng/mL, respectively. In the spiking test, the recovery of VD_3_ was 97.72%, and the relative standard deviation (RSD) value was 1.78% (Table 1).

The VD_3_ content of Penaeus sinensis by-products and other aquatic products was determined using Fe_3_O_4_@PPy composites-based extraction coupled with HPLC detection. The results in Table 2 show significant variations in VD_3_ content for the various species and parts tested in this study. Similarly, some authors have also reported significant differences in VD_3_ content for fish, both between species and within species [6]. For example, the average content of VD_3_ in mahi-mahi was only 1.11 μg/100 g, while it reached 45.3 μg/100 g in tilapia [34]. Baltic salmon had significantly higher VD_3_ content (26.5 μg/100 g) than farmed Norwegian salmon (5.9 μg/100 g) [6]. In this study, low average VD_3_ content was detected in squid (*Loliolus japonica*) meat (2.86 μg/100g) and in silver pomfret (*Pampus argenteus*) (4.65 μg/100 g). By comparison, the tested clams *Paphia undulata*, *Cyclina sinensis*, and razor clam (*Sinonovacula constricta*) had high VD_3_ content (>70 μg/100 g), suggesting these shellfish are a good source of dietary VD_3_. Furthermore, it was noted that the average VD_3_ content in the by-products of Penaeus sinensis (*Solenocera crassicornis*), Pacific white shrimp (*Litopenaeus vannamei*), and cuttlefish (*Sepia esculenta*) was higher than 10 μg/100 g, which exceeds the recommended daily intake of 5 μg/d [6]. In addition, the content of VD_3_ was higher in the by-products than that the corresponding muscle tissue (*p* < 0.05). These findings also suggest that the by-product of Pacific white shrimp should be considered a good raw material for VD_3_ extraction.

## 4. Conclusions

In the present work, we fabricated an effective adsorbent composed of Fe_3_O_4_ NPs functionalized with a PPy coating for VD_3_ extraction. The results of SEM, TEM, XRD, FTIR and TGA prove that the Fe_3_O_4_@PPy composites have a core-shell structure. The adsorption of VD_3_ from saponified Penaeus sinensis by-products to Fe_3_O_4_@PPy composites was optimal under the following conditions: pH 9.0 with a 25 mg dose at 25 °C and 11 min adsorption time. The adsorbed VD_3_ could be effectively desorbed from the binding sites of the Fe_3_O_4_@PPy composites with methanol after static contact for 3 min. The accuracy and reproducibility of the developed method for VD_3_ extraction and detection were quite satisfactory as evidenced by a high linear correlation coefficient and a low intra-day RSD. Compared to other conventional methods, the proposed method is more rapid since it does not require complicated extraction and concentration procedures. Instead, VD_3_ can be separated from complex samples within minutes by quick and easy magnetic separation. Furthermore, this method is environmentally friendly since it requires less organic solvent. In addition, the results for VD_3_ content in the tested aquatic products will provide important reference information for the rational selection of products for the development of VD_3_-fortified foods.

## Figures and Tables

**Table 1:** Quality control parameters for the developed method for determining VD_3_ content in saponified Penaeus sinensis by-products (*n* = 6).

Linearity Range (μg/mL)	r^2^	LOD (ng/mL)	LOQ (ng/mL)	Recovery (%)	RSD%
0.100–10.0	0.9989	10	33	97.72	1.78

**Table 2:** Average content of VD_3_ in the examined aquatic products.

Species	VD_3_ (μg/100 g)	
	Mean *	SD
Penaeus sinensis (*Solenocera crassicornis*) (by-products)	12.12 ^e^	1.39
Pacific white shrimp (*Litopenaeus vannamei*) (muscle tissue)	10.77 ^f^	2.06
Pacific white shrimp (*Litopenaeus vannamei*) (by-products)	29.34 ^c^	0.36
Cuttlefish (*Sepia esculenta*) (muscle tissue)	10.56 ^g^	0.19
Cuttlefish (*Sepia esculenta*) (by-products)	10.94 ^f^	0.52
Squid (*Loliolus japonica*) (muscle tissue)	2.86 ^i^	0.63
Squid (*Loliolus japonica*) (by-products)	6.44 ^h^	0.20
Clam (*Cyclina sinensis*)	80.81 ^b^	10.39
Clam (*Paphia undulata*)	131.76 ^a^	3.72
Razor clam (*Sinonovacula constricta*)	73.77 ^b^	6.38
Chlamys farrer (*Azumapecten farreri*)	23.10 ^d^	0.86
Silver pomfret (*Pampus argenteus*)	4.65 ^h,i^	2.84

Note: “*” represents the VD_3_ content, expressed as the mean ± standard deviation (SD) (*n* = 3). Different letters (a–i) against the mean content of VD_3_ for each species suggest a significant difference (*p* < 0.05).

## Data Availability

The data presented in this study are available on request from the corresponding author upon reasonable request.

## References

[1] Holick M.F. (2007). Vitamin D deficiency. N. Engl. J. Med..

[2] Reis A.F., Hauache O.M., Velho G. (2005). Vitamin D endocrine system and the genetic susceptibility to diabetes, obesity and vascular disease. A review of evidence. Diabetes Metab..

[3] Zhang M., Li P., Zhu Y., Chang H., Wang X., Liu W., Zhang Y., Huang G. (2015). Higher visceral fat area increases the risk of vitamin D insufficiency and deficiency in Chinese adults. Nutr. Metab..

[4] Maurya V.K., Aggarwal M. (2019). A phase inversion based nanoemulsion fabrication process to encapsulate vitamin D_3_ for food applications. J. Steroid Biochem..

[5] Bartoluccia G., Giocaliere E., Boscaro F., Vannacci A., Gallo E., Pieraccini G., Moneti G. (2011). Vitamin D_3_ quantification in a cod liver oil-based supplement. J. Pharm. Biomed. Anal..

[6] Malesa-Ciećwierz M., Usydus Z. (2015). Vitamin D: Can fish food–based solutions be used for reduction of vitamin D deficiency in Poland?. Nutrition.

[7] Hua M.Z., Feng S., Wang S., Lu X. (2018). Rapid detection and quantification of 2,4-dichlorophenoxyacetic acid in milk using molecularly imprinted polymers–surface–enhanced Raman spectroscopy. Food Chem..

[8] Mao X., Wan Y., Li Z., Chen L., Lew H.L., Yang H. (2020). Analysis of organophosphorus and pyrethroid pesticides in organic and conventional vegetables using QuEChERS combined with dispersive liquid-liquid microextraction based on the solidification of floating organic droplet. Food Chem..

[9] Enko D., Kriegshäuser G., Stolba R., Worf E., Halwachs-Baumann G. (2015). Method evaluation study of a new generation of vitamin D assays. Biochem. Med..

[10] Jiao Z., Zhang Y., Fan H. (2016). Ultrasonic-microwave method in preparation of polypyrrole-coated magnetic particles for vitamin D extraction in milk. J. Chromatogr. A.

[11] Jamal F., Shivam P., Kumari S., Singh M.K., Sardar A.H., Murugesan S., Narayan S., Gupta A.K., Pandey K., Das V.N.R. (2017). Identification of Leishmania donovani antigen in circulating immune complexes of visceral leishmaniasis subjects for diagnosis. PLoS ONE.

[12] Tolmacheva V.V., Apyari V.V., Furletov A.A., Dmitrienko S.G., Zolotov Y.A. (2016). Facile synthesis of magnetic hypercrosslinked polystyrene and its application in the magnetic solid–phase extraction of sulfonamides from water and milk samples before their HPLC determination. Talanta.

[13] Demirer G.S., Okur A.C., Kizilel S. (2015). Synthesis and design of biologically inspired biocompatible iron oxide nanoparticles for biomedical applications. J. Mater. Chem. B.

[14] Thinh N.N., Hanh P.T.B., Ha L.T.T., Anh L.N., Hoang T.V., Hoang V.D., Dang L.H., Van Khoi N., Lam T.D. (2013). Magnetic chitosan nanoparticles for removal of Cr (VI) from aqueous solution. Mater. Sci. Eng. C.

[15] Yu X., Yang H.S. (2017). Pyrethroid residue determination in organic and conventional vegetables using liquid-solid extraction coupled with magnetic solid phase extraction based on polystyrene-coated magnetic nanoparticles. Food Chem..

[16] Yu X., Li Z., Zhao M., Lau S.C.S., Tan H.R., Teh W.J., Yang H., Zheng C., Zhang Y. (2019). Quantification of aflatoxin B_1_ in vegetable oils using low temperature clean-up followed by immuno-magnetic solid phase extraction. Food Chem..

[17] Shalali F., Cheraghi S., AliTaher M. (2022). A sensitive electrochemical sensor amplified with ionic liquid and N-CQD/Fe_3_O_4_ nanoparticles for detection of raloxifene in the presence of tamoxifen as two essentials anticancer drugs. Mater. Chem. Phys..

[18] Zhao H.Y., Huang M.Y., Wu J.R., Wang L., He H. (2016). Preparation of Fe_3_O_4_@PPy magnetic nanoparticles as solid-phase extraction sorbents for preconcentration and separation of phthalic acid esters in water by gas chromatography–mass spectrometry. J. Chromatogr. B.

[19] Zhang Z.M., Zhu L., Ma Y.J., Huang Y.C., Li G.K. (2013). Preparation of polypyrrole composite solid-phase microextraction fiber coatings by sol-gel technique for the trace analysis of polar biological volatile organic compounds. Analyst.

[20] Zhou J., Lü Q.F., Luo J.J. (2017). Efficient removal of organic dyes from aqueous solution by rapid adsorption onto polypyrrole–based composites. J. Clean. Prod..

[21] Chithra K., Akshayaraj R.T., Pandian K. (2018). Polypyrrole-Protected Magnetic Nanoparticles as an Excellent Sorbent for Effective Removal of Cr(VI) and Ni(II) from Effluent Water: Kinetic Studies and Error Analysis. Arab. J. Sci. Eng..

[22] Gao Q., Luo D., Bai M., Chen Z., Feng Y. (2011). Rapid Determination of Estrogens in Milk Samples Based on Magnetite Nanoparticles/Polypyrrole Magnetic Solid-Phase Extraction Coupled with Liquid Chromatography–Tandem Mass Spectrometry. J. Agric. Food Chem..

[23] Zhang M., Lian K., Ai L., Kang W., Zhao T. (2020). Simultaneous determination of 11 antiseptic ingredients in surface water based on polypyrrole decorated magnetic nanoparticles. RSC Adv..

[24] Strobel N., Buddhadasa S., Adorno P., Stockham K., Greenfield H. (2013). Vitamin D and 25-hydroxyvitamin D determination in meats by LC-IT-MS. Food Chem..

[25] De Azevedo A.M., Losada A.P., Ferreiro I., Riaza A., Losada V., Russo T., Boglione C., Vázquez S., Quiroga M.I. (2021). Skeletal Anomalies in Senegalese Sole (*Solea senegalensis*, Kaup) Fed with Different Commercial Enriched Artemia: A Study in Postlarvae and Juveniles. Animals.

[26] Nalle C., Wahid F., Wulandari R.O.I., Sabarudin A. (2019). Synthesis and characterization of magnetic Fe_3_O_4_ nanoparticles using oleic acid as stabilizing agent. Rasayan J. Chem..

[27] Li X.H., Yin Z.D., Zhai Y.J., Kang W.J., Shi H.M., Li Z.N. (2020). Magnetic solid–phase extraction of four β–lactams using polypyrrole-coated magnetic nanoparticles from water samples by micellar electrokinetic capillary chromatography analysis. J. Chromatogr. A.

[28] Fard S.M., Ahmadi S.H., Hajimahmodi M., Fazaeli R., Amini M. (2020). Preparation of magnetic iron oxide nanoparticles modified with imidazolium-based ionic liquids as a sorbent for the extraction of eight phthalate acid esters in water samples followed by UPLC-MS/MS analysis: An experimental design methodology. Anal. Methods.

[29] Tamaura Y., Buduan P.V., Katsura T. (1981). Studies on the oxidation of iron(II) ion during the formation of Fe_3_O_4_ and α-FeO(OH) by air oxidation of Fe[OH]_2_ suspensions. J. Chem. Soc. Dalton Trans..

[30] Chen W., Li X.W., Xue G., Wang Z.Q., Zou W.Q. (2003). Magnetic and conducting particles: Preparation of polypyrrole layer on Fe_3_O_4_ nanospheres. Appl. Surf. Sci..

[31] Bhaumik M., Leswifi T.Y., Maity A., Srinivasu V.V., Onyango M.S. (2011). Removal of fluoride from aqueous solution by polypyrrole/Fe_3_O_4_ magnetic nanocomposite. J. Hazard. Mater..

[32] Jakobsen J., Knuthsen P. (2014). Stability of vitamin D in foodstuffs during cooking. Food Chem..

[33] Jakobsen J., Saxholt E. (2009). Vitamin D metabolites in bovine milk and butter. J. Food Compos. Anal..

[34] Bilodeau L., Dufresne G., Deeks J., Clément G., Bertrand J., Turcotte S., Robichaud A., Beraldin F., Fouquet A. (2011). Determination of vitamin D_3_ and 25-hydroxyvitamin D_3_ in foodstuffs by HPLC UV-DAD and LC–MS/MS. J. Food Compos. Anal..

